# Next-gen sequencing identifies non-coding variation disrupting miRNA-binding sites in neurological disorders

**DOI:** 10.1038/mp.2017.30

**Published:** 2017-03-14

**Authors:** P Devanna, X S Chen, J Ho, D Gajewski, S D Smith, A Gialluisi, C Francks, S E Fisher, D F Newbury, S C Vernes

**Affiliations:** 1 Neurogenetics of Vocal Communication Group, Max Planck Institute for Psycholinguistics, Nijmegen, The Netherlands; 2 Language and Genetics Department, Max Planck Institute for Psycholinguistics, Nijmegen, The Netherlands; 3 Department of Developmental Neuroscience, Munroe Meyer Institute, University of Nebraska Medical Center, Omaha, NE, USA; 4 Department of Translational Research in Psychiatry, Max Planck Institute of Psychiatry, Munich, Germany; 5 Donders Institute for Brain, Cognition and Behaviour, Nijmegen, The Netherlands; 6 Wellcome Trust Centre for Human Genetics, University of Oxford, Oxford, UK; 7 Department of Biological and Medical Sciences, Faculty of Health and Life Sciences, Oxford Brookes University, Oxford, UK

## Abstract

Understanding the genetic factors underlying neurodevelopmental and neuropsychiatric disorders is a major challenge given their prevalence and potential severity for quality of life. While large-scale genomic screens have made major advances in this area, for many disorders the genetic underpinnings are complex and poorly understood. To date the field has focused predominantly on protein coding variation, but given the importance of tightly controlled gene expression for normal brain development and disorder, variation that affects non-coding regulatory regions of the genome is likely to play an important role in these phenotypes. Herein we show the importance of 3 prime untranslated region (3'UTR) non-coding regulatory variants across neurodevelopmental and neuropsychiatric disorders. We devised a pipeline for identifying and functionally validating putatively pathogenic variants from next generation sequencing (NGS) data. We applied this pipeline to a cohort of children with severe specific language impairment (SLI) and identified a functional, SLI-associated variant affecting gene regulation in cells and post-mortem human brain. This variant and the affected gene (*ARHGEF39*) represent new putative risk factors for SLI. Furthermore, we identified 3′UTR regulatory variants across autism, schizophrenia and bipolar disorder NGS cohorts demonstrating their impact on neurodevelopmental and neuropsychiatric disorders. Our findings show the importance of investigating non-coding regulatory variants when determining risk factors contributing to neurodevelopmental and neuropsychiatric disorders. In the future, integration of such regulatory variation with protein coding changes will be essential for uncovering the genetic causes of complex neurological disorders and the fundamental mechanisms underlying health and disease.

## Introduction

Neuropsychiatric and neurodevelopmental disorders affect more than 15% of the population^1^ and span a range of complex phenotypes including schizophrenia (SCZ), autism spectrum disorder (ASD), intellectual disability, bipolar disorder, attention deficit hyperactivity disorder (ADHD), and language/communicative impairments. Great strides have been made in understanding the genetic factors contributing to these disorders by screening large cohorts of affected individuals using next generation sequencing (NGS) approaches such as whole-exome sequencing (WES) and more recently, whole-genome sequencing (WGS). However, for many neuropsychiatric disorders, the underlying genetic causes can be convoluted sometimes involving rare variation across a large number of causative genes, small contributions from common variants or no clear genetic causes despite high heritability of the disorder. As such, neuropsychiatric and neurodevelopmental disorders currently suffer from a high degree of missing heritability that demands new and different approaches when attempting to identify and assess genetic variation that could contribute to these disorders.

WES has been a recent method of choice for uncovering causes of neuropsychiatric disorders such as intellectual disability and ASD^2, 3, 4, 5^ due to its ability to rapidly and cheaply survey the protein coding portions of the genome. However, by design WES only surveys around 1% of the total genome sequence meaning that the majority of genomic variation in each individual cannot be assessed via this method. The importance of surveying non-protein coding DNA—which makes up ~99% of the human genome—is becoming apparent with the growing popularity of WGS studies. WGS has now successfully been used to identify hot-spots in the genome that are particularly susceptible to *de novo* mutations,^6^ mutation parent-of-origin effects,^7^ novel genes underlying intellectual disability^8^ and the prevalence of disruptions of non-coding regulatory regions in autistic individuals.^9, 10, 11^ These studies make it clear that we must take the contribution of non-coding variation seriously if we are to fully understand the genetics of neuropsychiatric and neurodevelopmental disorders.

Within non-coding regions of the human genome, regulatory elements such as promoters, enhancers and untranslated regions are strong candidate regions for pathogenic variation because they direct how much, when and where a gene is expressed. Variation in regulatory regions such as single nucleotide variants (SNV’s) or small indels, as well as larger scale copy number variants have all been shown to significantly affect gene expression.^12, 13, 14, 15^ Regulatory variants that result in significantly reduced gene expression could mimic loss of function coding mutations. Conversely, given that for many genes too much expression or expression at the wrong place or time can be as detrimental as gene loss, mutations that increase gene expression or change its timing/location can also contribute to disorder.^13, 16, 17^ Altered gene expression patterns are a common feature of neuropsychiatric disorders such as ASD and SCZ and in some cases these changes in gene expression can be linked directly to genomic/pathogenic variation.^12, 18, 19, 20^ This evidence points to a strong link between aberrant gene expression and neurological disorders, suggesting that variants in non-coding regulatory elements are excellent candidates to fill in some of the missing heritability of neuropsychiatric and neurodevelopmental disorders.

To this end, we chose to investigate non-coding variation in an NGS cohort, focusing specifically on a well-characterised regulatory region that strongly influences gene expression: the 3 prime untranslated region (3′UTR). 3′UTRs of genes have long been considered likely candidates for pathogenic mutations^21, 22, 23, 24^ and have been implicated in a range of neurological disorders. Sequencing of genes involved in tinnitus, Parkinson’s disease, Tourette’s syndrome and ASD have linked common single-nucleotide polymorphisms (SNPs) and rare variants in 3′UTR regions to these disorders.^17, 23, 24, 25^ In addition, a number of key considerations make these regions strong candidates for pathogenicity and highly amenable to study. First, 3′UTR regions are found at the end of all protein coding genes in the genome and are comprehensively annotated^26^ making their identification routine. Second, 3′UTR regions directly affect the amount of protein produced from a gene transcript via interactions with small regulatory RNA molecules known as microRNAs. Because these microRNA interactions are based on complementary nucleotide pairing with short sequences (7–20 bp) in the 3′UTR regions, they can be accurately predicted from sequence data in a high throughput manner. Finally, the functional effects of observed variants can be tested in the lab using simple, scalable assays. Functional testing to show that putative causative variants have cellular consequences is crucial for determining pathogenicity of both coding and non-coding variation and the potential scalability of these assays makes testing microRNA binding site (MBS) variants particularly attractive for large-scale NGS studies.

Given all these factors, we designed an approach for using NGS data to identify and functionally test the effects of variation in the 3′UTRome. Herein we show the efficacy of this method using WES data from a cohort of children classified as having a neurodevelopmental disorder: specific language impairment (SLI). SLI, the failure to acquire age appropriate language skills in the absence of any explanatory factors (e.g. intellectual disability), affects up to 8% of school age children.^27, 28^ Despite strong evidence for genetic underpinnings of language impairment, efforts to identify causative factors via linkage and association studies have found multiple genomic regions, all predicted to have small effects.^29, 30, 31^ Thus, like many other neurodevelopmental disorders, SLI seems to have a heterogeneous set of genetic risk factors contributing to the phenotype. We used WES data from a cohort of 43 affected children with severe SLI^32^ to demonstrate that it is possible to identify putative causative variants in non-coding 3′UTR regions using NGS data sets. Importantly we showed that variants can have direct functional consequences for gene expression by affecting miRNA binding sites, making them strong candidates for pathogenicity. To show the wider relevance of this class of variation we demonstrated that non-coding 3′UTR variants that disrupt miRNA binding sites are found across a range of neurological phenotypes, emphasising their potential relevance to neuropsychiatric and neurodevelopmental disorders.

## Materials and methods

### Variant identification in SLI cohort

Full methods on WES sequencing and variant calling are described by Chen *et al.*^32^ Briefly, for sequencing, the exome was captured by SureSelect Human All Exon version-2 50 Mb kit (Agilent, Santa Clara, CA, USA), sequenced using the SOLiD series 5500xl DNA sequencing platform (Life Technologies, Carlsbad, CA, USA) and called via the standard BWA-GATK pipeline, followed by quality filtering as described by Chen *et al.*^32^ Ethical agreement and informed consent from participants were obtained as described previously.^29^

Genome-wide coordinates (hg19) for MBSs were downloaded from the Targetscan 6.2 database.^33^ To identify WES variants that were overlapping with these microRNA sites we used the BEDTools intersectBED function.^34^

### Data availability

The primary data for the Chen *et al.*^32^ SLI exome study are deposited at The Language Archive (TLA: https://corpus1.mpi.nl/ds/asv/?0), a public data archive hosted by the Max Planck Institute for Psycholinguistics. Data are stored at the TLA under the node ID: MPI2010433#, and accessible with a persistent identifier: https://hdl.handle.net/1839/00-0000-0000-001E-AD41-2@view. Access can be granted upon request. TLA content is also visible from the Data Archiving and Networked Services (DANS) database, the Dutch national organization for sustained access to digital research data.

### Cloning of constructs for functional assays

To generate the microRNA expression constructs for miR-215, -342 and -346, regions encoding the primary transcripts were PCR amplified using the primers listed in Supplementary Table S3 ('miRNAs'). The PCR products were then cloned using *Age*I and *Eco*RI restriction sites in the pLKO.1 expression vector (Invitrogen, Carlsbad, CA, USA) and the sequences were confirmed by Sanger sequencing.

miR-433 was initially cloned in the same way, but this construct did not express functional miR-433 when tested in cells (data not shown). As such an alternative approach was undertaken. MicroRNA primary transcripts have been successfully used to drive expression of synthetic short hairpin RNA (shRNA) to facilitate gene knockdown.^35^ In such hybrid constructs, the sequence of the shRNA replaces the sequence that creates the stem–loop of the endogenous miR. Because we knew that miR-342 was successfully expressed (Supplementary Figure S1C), we inserted the stem–loop of miR-433 (which is responsible for generating the mature miR-433 sequence) into a plasmid already containing an expression cassette for miR-342. To facilitate removal of the miR-342 stem–loop from the miR-342 expression vector unique *Xba*I and *Sal*I restriction sites were engineered at the 5′ and 3′ (respectively) of the miR-342 stem–loop. The miR-433 stem–loop was amplified using primers containing *Xba*I and *Sal*I restriction sites (detailed in Supplementary Table S3) and the PCR product was cloned in place of the miR-342 stem–loop using *Xba*I and *Sal*I. The sequence was confirmed via Sanger sequencing. Expression and functionality of this mir-433 construct was confirmed in a 'positive control' reporter assay (see below; Supplementary Figure S1A).

'Positive control' reporter constructs were used to confirm that all cloned microRNAs were expressed and able to regulate gene expression by interacting with ideal target sites in a reporter assay (Supplementary Figure S1). Positive control reporters were generated as described previously.^36, 37^ Briefly, oligonucleotides containing two high-sensitivity binding sites for the cognate miRNAs were designed. To clone the reporter cassettes, sense and antisense oligonucleotides were generated and annealed to each other to form overhangs compatible with *Kfl*I restriction sites—allowing directional cloning into compatible sites. Oligonucleotides used for the generation of the reporters are listed in Supplementary Table S3 ('miRNA positive control reporters').

The 3′UTR regions for each candidate gene spanning the patient identified variants (approximately 300–400 bp; see Figure 3a) were cloned into the pGL4.24 luciferase expression vector (Promega, Madison, WI, USA) as reporter constructs. Control human genomic DNA (Novagen, EMD Millipore, Billerica, MA, USA) was used to PCR amplify the regions of interest (using the primers in Supplementary Table S3; '3′UTR'). The sequences were confirmed via Sanger sequencing and shown to contain the control/reference allele. PCR products were cloned downstream of the luciferase reporter gene using *Xba*I and *Fse*I restriction sites. Vectors carrying the alternative allele or with a deletion of the MBS were generated using the QuickChange Site-Directed Mutagenesis kit (Agilent Technologies) following the manufacturer's instructions and using the primers in Supplementary Table S3 ('3′UTR SDM'). The presence of the desired changes was confirmed via Sanger sequencing.

### Cell culture and transfection

We performed the reporter assays in human HEK293 cells and for comparison in a mouse neuronal cell line: Neuro2A (N2A) cells. Cell lines were obtained from ATCC (LGC Standards, Teddington, UK) and are routinely screened for mycoplasma contamination. Both cell lines are a suitable cellular model as they are easy to culture and reach very high transfection efficiency. The protein machinery that microRNAs use to regulate gene expression is ubiquitous and we and others have previously shown that this machinery is functional across different cell types.^36, 38, 39, 40^ All the experiments were carried out using cells grown in Dulbecco's modified Eagle's medium (Invitrogen) media supplemented with 10% fetal calf serum (Sigma-Aldrich, St Louis, MO, USA) and 2 mm penicillin/streptomycin. Cells were kept for the entire length of the experiments at 37 °C in the presence of 5% CO_2_. Transfections were performed using GeneJuice (Novagen) following the manufacturer's instructions.

### Luciferase assay

3.0 × 10^4^ cells were seeded in each well of a 24-well plate (60–70% confluence) 24 h before transfection. Reporter constructs were co-transfected into cells alongside the microRNA expression vector and a Renilla reporter (pRL-TK) for internal normalisation. Forty-eight hours post-transfection, firefly luciferase and Renilla luciferase activities were measured as per the manufacturer's instructions (Dual Luciferase reporter assay system; Promega).

### Western blotting

3.0 × 10^5^ HEK293 cells were seeded in each well of a six-well plate (60–70% confluence) 24 h prior to transfection of the microRNA (miR-215) expression vector (4 μg). Forty-eight hours post-transfection, cells were lysed in lysis buffer (0.1 m Tris, 150 mm NaCl, 10 mm EDTA, 0.2% Triton X-100, 1% PMSF, protease inhibitor cocktail) at 4 °C for 10 min and centrifuged at 10 000 *g* for 30 min at 4 °C, allowing cell debris to be pelleted and discarded. Western blotting was performed as described previously.^41^ Proteins were detected using primary antibodies for 60 min at room temperature or at 4 °C overnight. Secondary antibodies were applied for 30–60 min at room temperature. Antibodies were used as follows: ARHGEF39 (rabbit polyclonal, catalogue #131551, manufacturer NovoPro Bioscience, Shanghai, China) at 1/2000 concentration, B-actin (mouse monoclonal, catalogue #A5441, manufacturer Sigma-Aldrich) at 1/2000 concentration, secondary anti-mouse HRP-conjugated antibody (goat, catalogue #1706516, Bio-Rad Laboratories, Hercules, CA, USA) at 1/5000 concentration and secondary anti-rabbit HRP-conjugated antibody (donkey polyclonal, catalogue #16284, Abcam, Cambridge, UK) at 1/5000 concentration. Densitometry was performed using the Imagelab 5.2.1 (Bio-Rad) program to calculate arbitrary units reflecting relative protein expression levels for ARHGEF39 and β-actin.

### eQTL association studies

We utilised the Genotype-Tissue Expression project portal (GTEx) (http://www.gtexportal.org; GTEx Analysis Release V6p) to determine associations between individual SNPs and the expression of their corresponding protein coding genes. We assessed genes for single-tissue eQTL values and generated the multi-tissue eQTL comparison plot using the online ‘Gene Association’ tool. Plots for individual brain regions were generated using the ‘Test your own’ tool. Descriptions of the association and statistical analyses generated by GTEx can be found in the relevant references.^42, 43^ *P*-values are calculated for single-tissue associations and given that associations were generated for 44 tissues, using a stringent multiple testing correction (Bonferroni), *P*-values must be *P*<1.13e-3 to be considered significant.

### Variant identification in further NGS cohorts

We searched Pubmed for original research articles reporting human whole-genome or whole-exome data. Articles were selected that interrogated large cohorts related to neurodevelopmental and/or neuropsychiatric disorders, as well as parent-of-origin effects. Data sets could only be included for which variant call files including variants and genomic coordinates were made available online. The reported statistics from each research article were used to determine the number of potentially protein altering variants and classify these variants as exonic, frameshift, missense or nonsense changes. To determine the number of variants in MBSs, we used the BEDTools intersectBED function^34^ to overlap genomic coordinates of all reported variants with the genomic coordinates of all MBSs obtained from the Targetscan 6.2 database.^33^

## Results

### 3′UTR variants within MBSs can be identified from WES data

**Figure 1:**
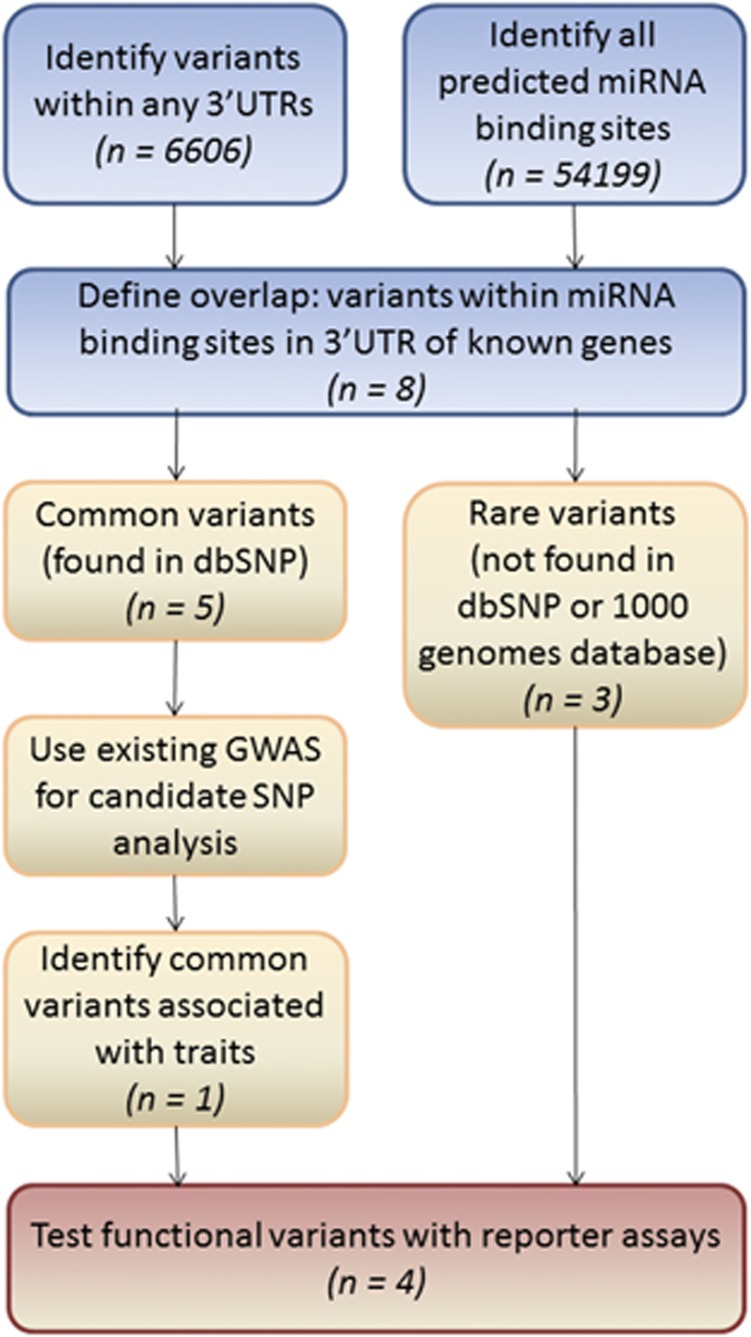
Workflow for identification of non-coding 3′UTRome variants in next generation sequencing (NGS) data. This flowchart demonstrates how variants can be identified in non-coding regulatory regions of the genome using NGS data. First, a list of all variants found within the 3′UTR regions of any genes was generated from WES data (*N*=6606). This was overlapped with all predicted microRNA-binding sites in the genome (via targetscan 6.2) (*N*=54 199) to create a list of variants that lie within miRNA binding sites in the 3′UTR of known genes (*N*=8). The identified variants fell into two categories; three rare variants found in a single proband and five common variants that were annotated in dbSNP. Common variants were further assessed using candidate SNP analysis in a GWAS study of a large SLI cohort. One of the common variants showed association with a quantitative measure of language impairment. SNVs that pass the bioinformatic screening (*N*=4) are characterised using reporter assays to demonstrate functionality of the wild-type site and consequences of patient identified variants. For each stage the number of variants/sites identified in each category in our SLI study is shown in brackets. 3'UTR, 3 prime untranslated region; GWAS, genome-wide association study; SLI, specific language impairment; SNP, single-nucleotide polymorphism; SNV, single-nucleotide variant; WES, whole-exome sequencing.

**Figure 2:**
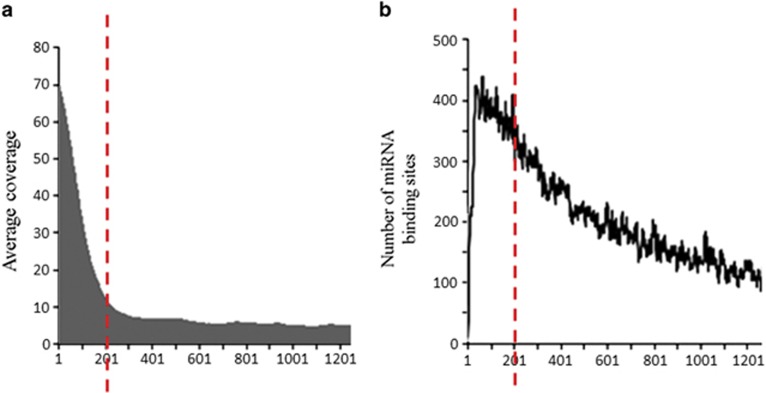
Identification of non-coding variants in exome sequencing data. (**a**) Average read depth profile across the 3′UTRome in the SLI WES data set. On average, we reached >10 × coverage for the first ~200 bp of the 3′UTR of a gene. For some genes, high coverage extended beyond this boundary, allowing variants to be called further along the 3′UTR region. (**b**) Distribution of all predicted microRNA-binding sites across the human 3′UTRome. In total, 54 199 miRNA-binding sites were predicted by Targetscan. These sites were mapped to their respective genomic positions, showing that 8127 sites were located within the first 200 bp of a 3′UTR region. This means that ~15% of all predicted miRNA-binding sites are located within the first 200 bp of the 3′UTR. This represents the region with the highest density of miRNA-binding sites in the 3′UTRome and is also the region for which we have sufficient depth of sequencing in the exome data to reliably call variation (see dotted red lines). 3'UTR, 3 prime untranslated region; SLI, specific language impairment; WES, whole-exome sequencing.

We designed a pipeline to identify and assess the functionality of both common and rare SNVs identified in non-coding 3′UTR regions of the genome and applied this to a WES data set from a cohort of 43 children with severe SLI^32^ (Figure 1). Chen *et al.*^32^ identified all SNVs present in the WES data throughout the genome. To rule out likely false positives, only SNVs with >10x sequence coverage were retained (see Supplementary Table S1 for SNV numbers identified in different regions of the genome in this data set). From all filtered variants we extracted only those SNVs that were within the 3′UTR region of a gene (*N*=6606, over 4651 3′UTR regions) from these data. For the majority of 3′UTRs in the genome (62.7% of 3′UTRs annotated by Ref-Seq), the first ~200 bp had a read depth ⩾10 allowing the reliable identification of variants in this region (Figure 2a). The highest density of MBSs is also found in this initial region of the 3′UTR (Figure 2b), suggesting that a large number of binding sites can be assessed per gene, even using the limited region of the 3′UTR covered by standard exome sequencing.

To determine if any of the 3′UTR variants fell within MBSs, we separately searched for all predicted sites using the TargetScan algorithm^33^ and overlaid these with the WES identified SNVs. Eight 3′UTR SNVs were thus identified within a sequence predicted to be bound by a microRNA. Three of these were rare variants, only found in a single individual and each in the 3′UTR of different genes: *BTN2A1*, *CENPJ* and *MTMR3* (Table 1). These were considered to be private mutations as they were not present in other data sets (dbSNP,^44^ 1000 genomes,^45^ ExAc browser^46^), indicating that they have a population frequency of less than 0.00082%. The presence of these rare variants in each of the relevant probands was confirmed with bi-directional Sanger sequencing (Supplementary Figure S2). Five of these SNVs were identified from dbSNP as SNPs found in the general population (frequency >0.1%); four are common (population frequency>1%) and one is rare (0.3%) (Table 1).

### A common variant within an MBS is associated with SLI

To determine the relevance of the common (SNP) variants to SLI we took advantage of genome-wide SNP data from the SLI Consortium (SLIC) sample. This data set comprised 285 SLI families recruited from five centres across the UK; The Newcomen Centre at Guy’s Hospital, London (now called Evelina Children’s Hospital); the Cambridge Language and Speech Project (CLASP); the Child Life and Health Department at the University of Edinburgh; the Department of Child Health at the University of Aberdeen; and the Manchester Language Study.^29, 31^ The 43 probands sequenced herein are a subset of this cohort. Two common SNPs (rs1054528 and rs190191374) were not genotyped in the SLIC (SLI Consortium) data set and thus could not be assessed. However, three of the five common SNPs were directly genotyped or imputed from the SLIC data set (rs72727021 (imputed), rs383362 (genotyped) and rs1049232 (genotyped)) and thus we could use these data for a candidate association analysis. Genotype data were available for 983 parents and children from these 285 SLI families. As previously described, various quantitative measures of language-related abilities were available for children in this cohort.^29, 47, 48^ Measures of expressive (ELS) and receptive (RLS) language were obtained using the Clinical Evaluations of Language Fundamentals (CELF-R).^49^ Verbal IQ (VIQ) and performance IQ (PIQ) were assessed using the Wechsler Intelligence Scale for Children^50^ and reading and spelling ability were measured with the Wechsler Objective Reading Dimensions (WORD).^51^ In addition, the phonological short-term memory of adults and children was assessed with a 28-item test of non-word repetition.^52^ Association was assessed within and between family units using the QFAM test in PLINK. This quantitative test of association employs an adaptive permutation procedure to account for the dependence between related individuals. One SNP (rs72727021), located in the 3′UTR of the *ARHGEF39* gene (also known as *C9orf100*), was associated with the non-word repetition measure in the SLI cohort (empirical *P*=7.7 × 10^−3^) (Supplementary Table S2). The alternative ‘C’ allele of rs72727021, which has a population frequency of 4.7% (10.8% in 120 CEPH individuals in 1000 genomes pilot phase I), is carried by 23% of the SLIC probands (MAF=12.3%) and is associated with a reduction of 10 points (0.66 s.d.) on the non-word repetition test in these individuals.

We sought to find supportive evidence for the association between rs72727021 and non-word repetition in other data sets; however, to our knowledge a replication data set does not exist. We nonetheless queried the association in two independent data sets: the Avon Longitudinal Study of Parents and Children (ALSPAC) cohort^53^ and the Colorado Learning Disabilities Research Centre (CLDRC) data set.^54^ Although no significant association was found in these data sets, a lack of support from these cohorts may reflect variations in sample ascertainment since the SLIC population was specifically selected for severe language phenotypes, or the differences in tests used to generate the language ability metrics. Further investigations in specifically selected samples will be needed to clarify the genetic association between rs72727021 and language phenotypes.

### Functional validation of a WES 3′UTR variant located within an MBS

**Figure 3:**
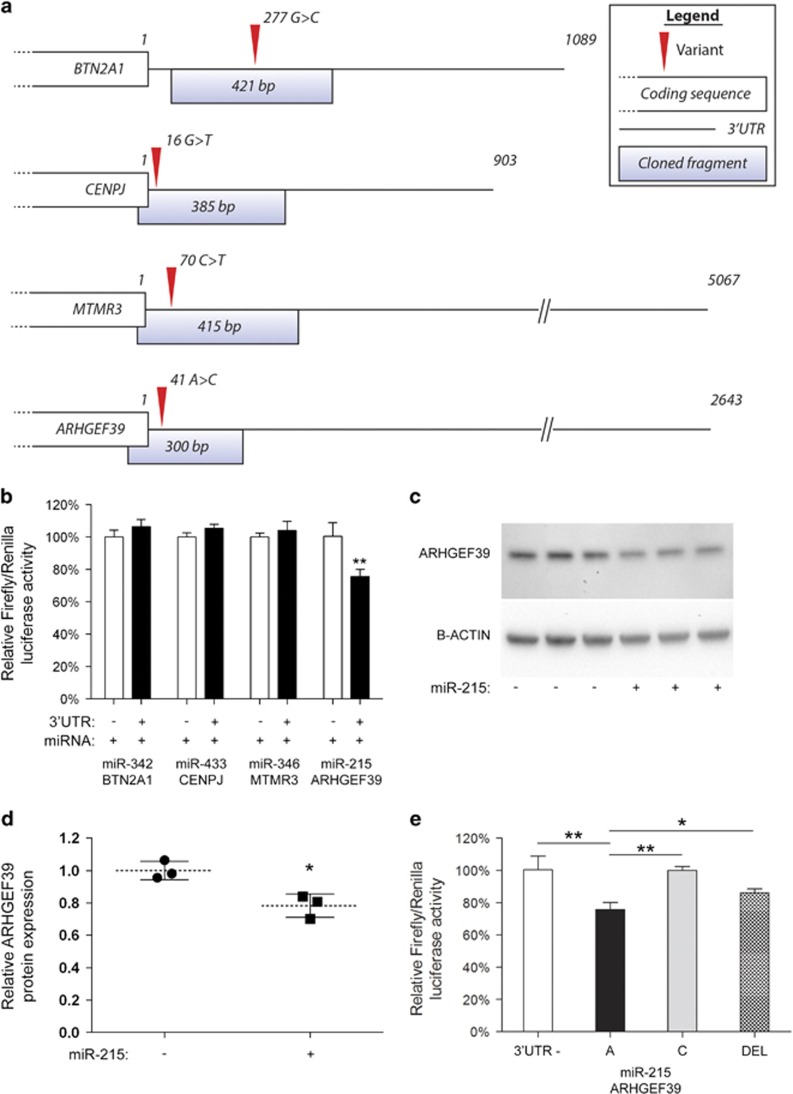
Functional consequences of 3′UTR variants identified in SLI exomes. (**a**) 3′UTR fragments of *BTN2A1*, *CENPJ*, *MTMR3* and *ARHGEF39* that spanned the predicted microRNA-binding sites were cloned downstream of the bioluminescent luciferase reporter gene. Schematic illustrates the length of the 3′UTR (depicted by a straight line) relative to the end of the protein coding sequence of each gene (depicted by a white box), the cloned fragments that was used in reporter assays, and the identity and position of the 3′UTR variants identified in the SLI cohort. (**b**) Luciferase reporter assays were used to demonstrate if predicted binding sites were functionally regulated by the predicted microRNAs. Candidate 3′UTR regions (shown in part **a**) were cloned downstream of the luciferase reporter gene. These UTR reporter constructs (3′UTR: +) were co-expressed with the microRNA that was predicted to regulate each UTR. To determine specificity of this regulation, control reporters were also tested that lacked any cloned fragment (3′UTR: −). Expression of the reporter gene was the same with (+) or without (−) the 3′UTR fragment for *BTN2A1*, *CENPJ* and *MTMR3*, showing that these sites were not regulated by these microRNAs (miR-342, miR-433 and miR-346). In contrast, reporter gene expression was significantly lower when the *ARHGEF39* 3′UTR fragment was present (+) compared to the reporter without any 3′UTR (−), showing that miR-215 represses gene expression by interacting with the 3′UTR when the reference allele 'A' is present. No differences in reporter activity were observed in the absence of miR-215 co-expression (data not shown). (**c**) miR-215 regulates endogenous *ARHGEF39*. Cells transfected with the miR-215 vector (miR-215; +) displayed lower expression of endogenous *ARHGEF39* than mock-transfected cells (miR-215; −), shown via western blotting. β-Actin was used as a loading control. Full scans of blots with ladders to indicate band size are given in Supplementary Figure S4. (**d**) Quantification of western blot data shown in part (**c**). The relative expression of *ARHGEF39* (normalised to β-actin) was calculated via densitometry and indicates significant repression of endogenous protein when miR-215 is present. (**e**) To determine if the presence of the alternative 'C' allele disrupts this regulatory relationship, we introduced the 'C' allele into the reporter gene 3′UTR. The presence of the SLI-associated 'C' allele abolished repression of the reporter gene by miR-215, showing its biological relevance. To show specificity of this effect the entire miR-215-binding site was deleted from the *ARHGEF39* 3′UTR reporter ('DEL') and this construct was also not regulated by miR-215. Deleting the entire miR-215 binding site ('DEL') was not significantly different to the effect of introducing the 'C' allele and neither of these were significantly different from the construct that had no 3′UTR cloned fragment present ('3′UTR −'). Significant differences between groups were calculated using an ANOVA test followed by *post hoc* Tukey calculation. Only statistically significant differences are noted. Significance is indicated by *P<0.05 and **P<0.01. All results are reported as the average±s.d. of three biological replicates. 3′UTR, 3 prime untranslated region; SLI, specific language impairment.

From our exome data we have identified four candidate variants located in MBSs that may be related to the SLI phenotype: one common variant (rs72727021) that is associated with non-word repetition in the SLI cohort and three rare (private) variants. Because these MBSs were identified via *in silico* predictions, we first set out to functionally validate the predicted interaction between these 3′UTR-binding sites and their cognate microRNAs using a reporter assay. We cloned an approx. 300–400 bp region from each genes 3′UTR, spanning the predicted miRNA-binding site (Figure 3a). This was inserted into an expression vector downstream of the luciferase reporter gene as part of its 3′UTR. We then determined the ability of the relevant microRNAs to regulate the predicted binding site within the 3′UTR of each gene. The binding sites identified within the 3′UTRs of *BTN2A1*, *CENPJ* and *MTMR3* were not regulated by their predicted microRNAs (miR-342, miR-433 and miR-346, respectively) in these reporter assays, suggesting they are not functional binding sites (Figure 3b). As such we conclude that the rare variants found within these binding sites are unlikely to disrupt microRNA-3′UTR interactions, and thus unlikely to directly contribute to SLI via this regulatory mechanism. However, 3′UTRs can have other functions, and as such we cannot rule out that the presence of these variants might affect other 3′UTR-dependent post-transcriptional processes and lead to SLI-related phenotypic outcomes.

The binding site within the 3′UTR of *ARHGEF39* is predicted to be recognised and bound by both miR-215 and miR-192. The seed sequence for these microRNAs are identical and both microRNAs have the same confidence score (as predicted by Targetscan), suggesting that they are equally able to bind this site. Given this equivalence, we used miR-215 to demonstrate the functionality of this site. The *ARHGEF39* 3′UTR reporter containing the reference allele of the rs72727021 SNP was significantly downregulated by miR-215 (Figure 3b). miR-215 was also able to significantly repress the expression of endogenous *ARHGEF39* as demonstrated via western blotting (Figures 3c and d). These data demonstrate that the MBS found in the 3′UTR of *ARHGEF39* is functional, and that miR-215 downregulates expression by interacting with this site when the reference 'A' allele is present.

We then went on to determine how the presence of the SLI-associated alternative 'C' allele affected this regulatory relationship. Introducing the alternative 'C' allele into the 3′UTR of the reporter construct abolished regulation by miR-215 (Figure 3e). To confirm the specificity of this interaction we also made a variant where the miR-215-binding site in the 3′UTR was completely deleted. As expected miR-215 could no longer regulate the reporter construct when the deletion was introduced (Figure 3e). Interestingly this loss of regulation was not significantly different to the control vector (lacking a 3′UTR) or the vector carrying the alternative 'C' allele of rs72727021 in its 3′UTR. To confirm that this regulation also occurred in a neuronal cell line and was not a cell-type-specific effect, we also performed these assays in Neuro2A cells and saw equivalent results (see Supplementary Figure S3).

Together this demonstrates that the *ARHGEF39* 3′UTR can be downregulated by miR-215 when the rs72727021 reference allele ‘A’ is present. If the SLI-associated 'C' allele is present, miR-215 regulation is abolished leading to higher expression, and this effect is as severe as a complete deletion of the miR-215-binding site.

### *In vivo* brain expression differences are associated with rs72727021

**Figure 4:**
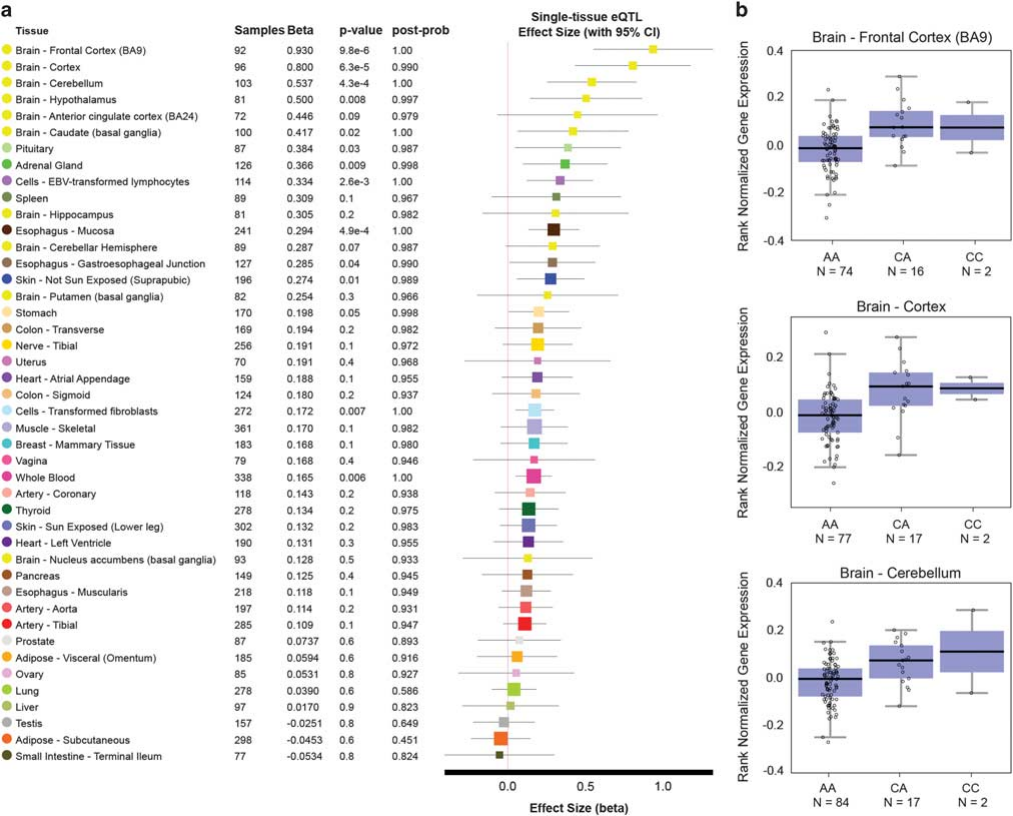
rs72727021 is associated with ARHGEF39 expression in the human brain. (**a**) A multi-tissue eQTL comparison was made for rs72727021 and ARHGEF39 using the GTEx database gene association tool across 44 tissue types. For each tissue is shown: the number of RNA-Seq samples with genotype (‘Samples’), the effect size (‘Beta’), the significance for that tissue (‘*P*-value’) and the posterior probability likelihood that an effect exists in each tissue in the cross-tissue meta-analysis (‘post-prob’). Tissues are listed by effect size and those with a post-prob >0.9 and *P*-value below *P*<1.13e-3 (to account for multiple testing correction) are considered likely to have an effect in that tissue and be significantly associated. (**b**) The three tissues with the most significant associations also had the highest effect sizes and all were found in the brain. Rank normalised gene expression for each of these tissues; frontal cortex (BA9), cortex and cerebellum are shown, plotting expression for each individual/sample with their corresponding genotype. In these three tissues, ARHGEF39 expression is higher when the rs72727021 alternative allele ('C') is present in the heterozygous (‘CA’) or homozygous (‘CC’) state.

In addition to these functional assays, we also identified strong support for the relevance of this variant in controlling *ARHGEF39* expression levels *in vivo*. We interrogated eQTL data from the GTEx portal^55^ across all 44 tissue/cell types present in the database (Figure 4a) and found that the rs72727021 SNP was significantly associated with *ARHGEF39* expression in the human brain. Individuals that were heterozygous or homozygous for the alternative ‘C’ allele had significantly higher *ARHGEF39* expression in regions of the brain (cortex *P*=6.3e-5; frontal cortex *P*=9.8e-6; cerebellum *P*=4.3e-4) (Figure 4b). This mirrors the loss of repression (higher reporter gene expression) we observed when the alternative 'C' allele was present (compared with the reference 'A' allele) during *in vitro* functional assays (Figure 3e). To support the specificity of this *in vivo* data we checked the GTEx portal for eQTL effects related to the other four common variants identified in this cohort (Table 1). No significant differences in expression were observed for these variants in the human brain, further supporting the significance of our findings.

Taken together, these findings show that we have identified a common non-coding SNP with a significant association to SLI and a functional effect on gene expression in both cell models and the human brain. We believe these data represent the first time an SLI-associated SNP has been reported to have direct functional consequences.

### Wider relevance of 3′UTRome variation to neurodevelopmental/neuropsychiatric disorders

Herein we designed and tested an approach that would allow the identification and functional validation of 3′UTRome variants from NGS data. We tested this approach using a cohort of language impaired children given the complex genetic underpinnings of this disorder. To illustrate the potential relevance of this approach for wider neurodevelopmental and neuropsychiatric disorders, we chose a selection of recent NGS studies searching for pathogenic or *de novo* variation in large cohorts and for which variant call files were made available online. These studies included whole-exome and WGS data sets from cohorts of bipolar disorder, SCZ, ASD and a study investigating parent-of-origin signatures of *de novo* mutations.^2, 7, 9, 11, 56, 57^ We determined the number of variants in 3′UTR regions that altered MBSs via intersection of these variants with Targetscan coordinates (as described above) and identified 37 MBS variants in total. In all studies tested at least one MBS variant was found, and up to 12 *de novo* variants could be identified per cohort (Table 2).

## Discussion

Herein we demonstrate an approach for identifying and validating the functionality of non-coding variants from NGS data. We used this approach to identify a non-coding SNP in an MBS that is associated with SLI and has functional consequences for gene expression. We also demonstrated the presence of such 3′UTR-MBS variants in wider cohorts of neuropsychiatric disorders including SCZ, ASD and bipolar disorder.

This work represents the first report of functional effects for a non-coding SNP associated with SLI and highlights a possible role for the rs72727021 variant and/or the *ARHGEF39* gene (in which the variant is found) in language disorder. Little is currently known regarding the *ARHGEF39* gene, but it is a member of the ARHGEF family of Rho guanine nucleotide exchange factors. These proteins act as molecular switches to regulate diverse processes including transcriptional regulation, cell migration, cell growth and dendritic development.^58, 59, 60^ Other members of this family have previously been implicated in language impairment (*ARHGEF19*),^29^intellectual disability (*ARHGEF6*)^61^ and moderate intellectual disability with speech delay (ARHGEF9).^4^ Future studies into the expression pattern and function of this gene may reveal a potential role for *ARHGEF39* in the development of language relevant circuitry in the brain.

Given the importance of non-coding DNA for regulating gene expression and the straightforward approach we propose for assessing these variants, we strongly recommend that analysis of 3′UTRome MBS variation be included routinely for all NGS data/pipelines. Although (by design) current WES platforms do not sequence the entire 3′UTR, we have shown that we can extract functional variants from exome sequence data using our SLI cohort. Furthermore, we have shown that other existing WES data sets investigating SCZ and ASD contain *de novo* variants in MBSs that may contribute to the aetiology of these neuropsychiatric disorders (Table 2). To date, it is estimated that more than one million exomes have been sequenced using WES. The methodology we propose here is immediately applicable to any of these existing WES data sets for which raw sequence reads or called variants are available. The widespread availability of WES data makes it a cost-effective and practical way to discover functionally relevant non-coding SNVs related to these neurological phenotypes.

Studies of complex disorders are increasingly making use of WGS, and including our pipeline when assessing WGS data from large cohorts will allow unprecedented identification of functional, non-coding risk factors. In the WGS data sets we surveyed (Table 2) even when cohorts were relatively small,^11, 56^ selection criteria were conservative (for example, only allowing *de novo* variants to be included^2, 7, 9, 11, 57^), or when variants were only included from a small number of candidate genes,^56^ at least one 3′UTR-MBS variant was identified per cohort, suggesting that this class of variants will be highly prevalent across WGS studies. Further supporting the relevance of these variants to phenotypes, the MBS variants we identified in these cohorts were often located in the 3′UTRs of genes previously implicated in their respective disorders. For example, in the bipolar cohort four MBS variants across six probands were identified in a leading candidate gene, *ANK3*.^56^ In ASD cohorts, MBS variants were identified in *BRD1*^2^ and *CHD2*,^9^ both of which have been previously linked to autism via identification of putatively pathogenic *de novo* protein-coding mutations and copy number variants.^62, 63, 64^ Indeed, taken together, these findings argue for examination of the 3′UTR regions of candidate genes across a range of disorders within existing NGS data sets. Our findings suggest it is likely that new 3′UTR-MBS variants will be identified via this approach that may help to explain some of the missing heritability of neurodevelopmental/neuropsychiatric disorders.

NGS has brought astonishingly rapid advances in the high throughput identification of variants related to phenotypes. Unfortunately our ability to determine which variants have functional consequences has not kept pace. Yet, if a genetic variant does not have molecular consequences for a gene or a cell, it is unlikely that it will be causative or pathogenic, making functional analysis crucial. Algorithms are widely used to predict functional/pathogenic consequences; however, these have very high false-positive/negative rates^65, 66^ reinforcing the importance of functional testing when attempting to link genetic variation to phenotypes. A major strength of our approach is that it provides a simple, high throughput method for validating the functional, molecular consequences of non-coding 3′UTRome variants. Because the functionality of a microRNA-3′UTR interaction can be determined via a simple set of reporter gene assays in any cell model, this approach facilitates high throughput and reliable assessment of the consequences of identified 3′UTR variants.

In summary, we report an accessible, rapid and biologically relevant method for assessing non-coding variation that adds significantly to our ability to identify causative variants from NGS data—a major challenge facing the future of genomics. Neuropsychiatric disorders suffer from a high degree of missing heritability and one of the ways we can address this issue is to look beyond a ‘protein-centric’ view of the genome and also address the relevance of non-coding variation. Adding this simple pipeline to the standard WES/WGS toolkit is expected to reveal a wealth of functional variation in largely overlooked non-coding regions of the genome and will help to identify new links between genes and disorders. Furthermore, by not stopping at identification but performing rigorous functional characterisation of the molecular consequences of these variants, we will shed new light on not only the causes of neurological disorders but the fundamental molecular mechanisms underlying health and disease.

## Figures and Tables

**Table 1:** SNVs located within predicted microRNA-binding sites in SLI probands

*Chr*	*Position*	*dbSNP*	*dbSNP Global MAF freq*	*Ref*	*Alt*	*Probands*	*Gene*	*microRNA*
6	26469054	—	—	G	C^a^	1	BTN2A1	miR-342
13	25457299	—	—	G	T^a^	1	CENPJ	miR-433
22	30421860	—	—	C	T^a^	1	MTMR3	miR-346
9	35661943	rs72727021	0.0469	A	C^a^	9	ARHGEF39	miR-192/215
16	87436764	rs1054528	NA	A^a^	G	42	MAP1LC3B	miR-204/211
16	79245820	rs383362	0.3223	G	T^a^	34	WWOX	miR-134/758
19	6751293	rs1049232	0.2404	T	G^a^	13	TRIP10	miR-17-5p
19	53086946	rs190191374	0.0030	G	T^a^	1	ZNF701	miR-199/199-5p

Abbreviations: SLI, specific language impairment; SNP, single-nucleotide polymorphism; SNV, single-nucleotide variant.

a Minor allele.

**Table 2:** Variants identified in a selection of NGS data sets

*Disorder*	*Study type*	*# subjects in cohort*	*Total # variants*	*# potentially protein altering*	*# within MBS*	*Gene(s)*	*Refs*
Bipolar disorder	WGS	99 probands	233	NA	4	ANK3 (candidate analysis)	^56^
SCZ	WES	623 trios	640	469^a^	1	NIPBL	^57^
ASD	WES	2500 families	5648	3283^a^	11	ANGEL2, PTPN14, ZBTB11, PAQR3, PCDHA1-13/ PCDHAC1-2, DDR1, CNPY3, PHF10, SF1, AGAP2, BRD1	^2^
ASD	WGS	53 families	7978	NA	3	TSLP, FOSL2, VAPA	^11^
ASD	WGS	200 trios	10387	193^b^	6	CTCF, CEP350, FUT11/CHCHD1, CHD2, EED, CUX1	^9^
Parent-of origin DNM	WGS	816 trios	35793	NA	12	PTPRJ, FUT4, NUP37, YLPM1, ZNF423, DKFZp434J194, CDC42EP1, CTNNA2, CLASP2, ADAM19, TUSC3 (2 hits)	^7^

Abbreviation: ASD, autism spectrum disorder; MBS, microRNA binding site; NA, not annotated; NGS, next generation sequencing; SCZ, schizophrenia; WES, whole-exome sequencing; WGS, whole-genome sequencing.

a Number classified as frameshift, missense or nonsense changes.

b Number classified as exonic.
