# Supplementary material for: Next-gen sequencing identifies non-coding variation disrupting miRNA-binding sites in neurological disorders
Source: Mol Psychiatry. 2017 Mar 14;23(5):1375–84. doi: 10.1038/mp.2017.30 (PMC5474318; doi:10.1038/mp.2017.30)
Supplement: Supplementary Information [file mp201730x1.docx]

**SUPPLEMENTARY DATA**

Description: Supplementary figures include reporter validation of functional miRNA’s (Figure S1), Sanger sequencing confirmation of rare variants (Figure S2), functional testing in an alternative cell line (Figure S3) and full scans of western blots from main Figure 3 to show protein size (Figure S4). Tables include SNV’s identified in the SLI cohort (Table S1), full metrics for the candidate association study (Table S2) and primers used for cloning or mutagenesis of reporter constructs (Table S3).

**SUPPLEMENTARY FIGURES**

**Figure S1. Reporter assays demonstrate the microRNA expression constructs produce functional microRNAs that repress reporter gene expression**. Luciferase reporter gene constructs were generated containing multiple repetitions of ideal binding sites for each microRNA downstream the gene coding region (as an artificial 3'UTR). The artificial 3'UTRs specific to miR-215, -342, and -433 contained 4 repetitions of the binding site each, while the artificial 3'UTR for miR-346 contained 6 repetitions of the binding site. These were used to demonstrate functionality of our cloned microRNAs. None of the artificial 3'UTRs were strongly regulated without the transfection of exogenous microRNA constructs, suggesting that the microRNA under study (miR-433, miR-346, miR-342 and miR-215) are either not endogenously expressed or only expressed at extremely low levels in our cell model (HEK293 cells). Following the addition of each microRNA expression vector, reporter gene expression was strongly and significantly downregulated, demonstrating that our microRNA constructs are highly expressed and functional. Significant differences between groups (no reporter vs miR-reporter for each condition) were calculated using students t-test. Significance is indicated by *p < 0.01 and ** p < 0.0001. All results are reported as the average +/− standard deviation of 3 biological replicates.


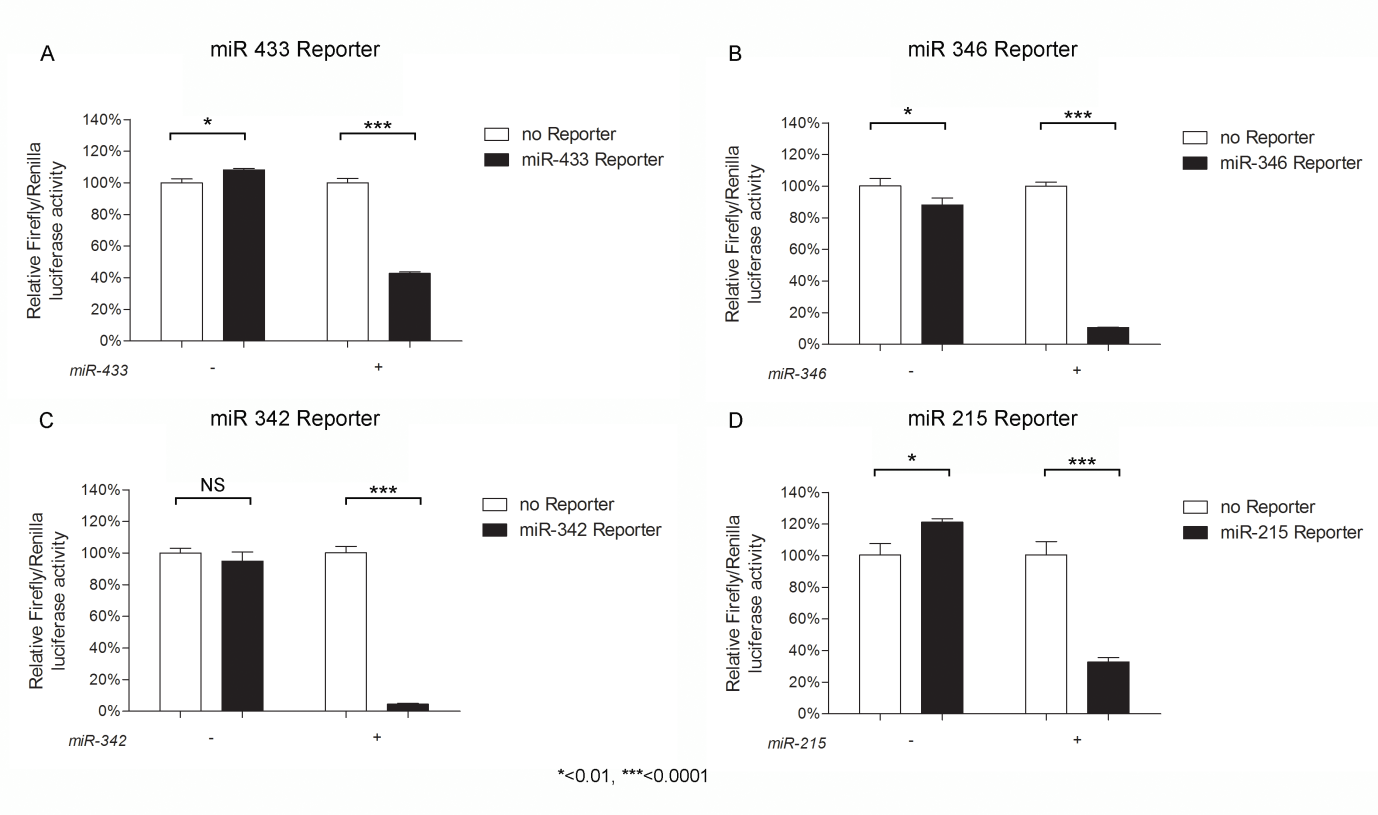


**Figure S2. Sanger sequencing confirmed the presence of the rare variants in the relevant probands.** The chromatogram shows the base found at each position in the probands' genome. Each proband carries the variant as it was identified in the exome sequencing.
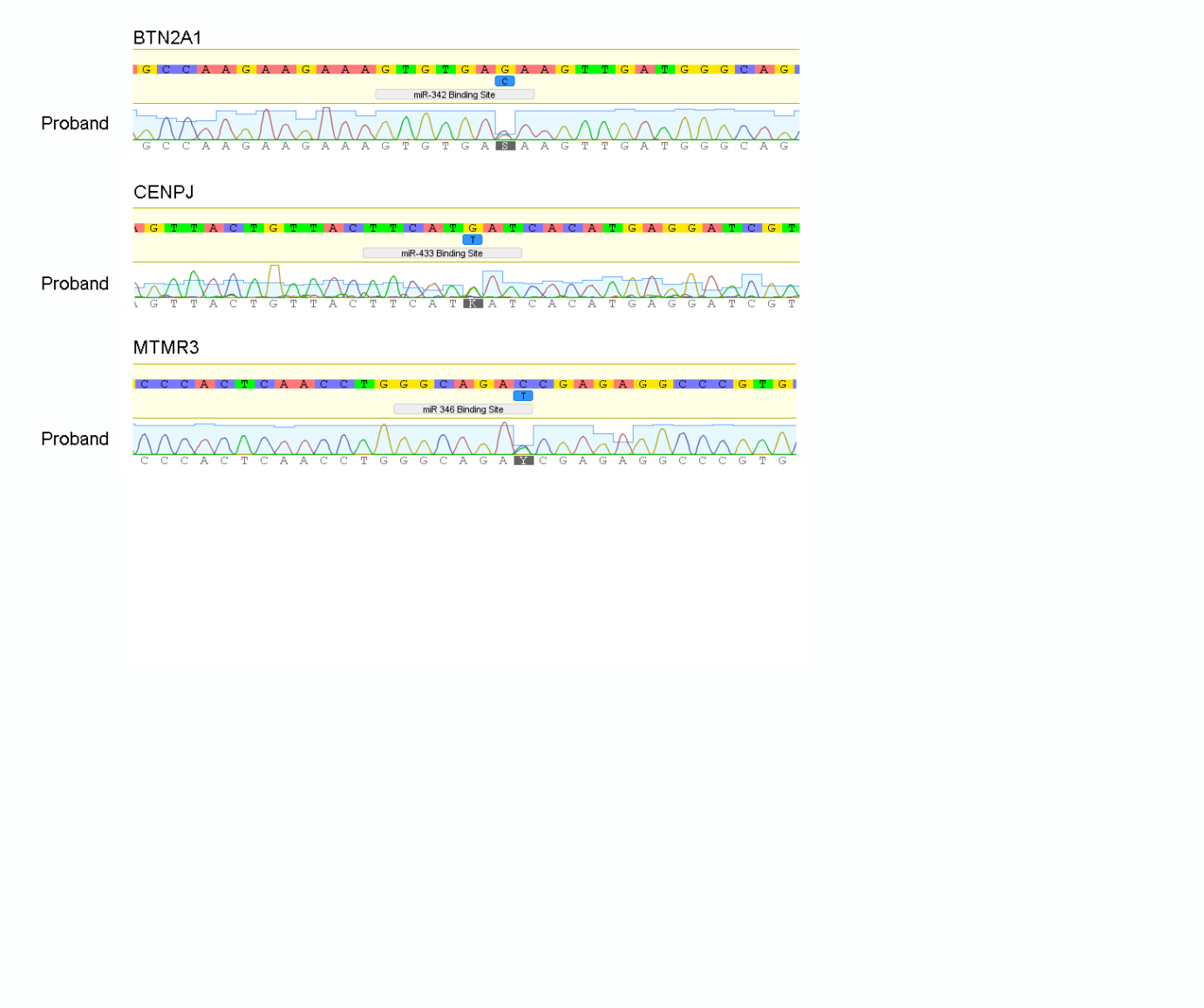


**Figure S3. Functional testing of *ARHGEF39* 3’UTR regulation in Neuro2A cells.** Luciferase reporter assays shown in Figure 3 (main text) were repeated in Neuro2A cells to demonstrate that these effects occurred in a neuronal cell line and were not cell type specific. As before, reporter gene expression was significantly lower when the *ARHGEF39* 3'UTR fragment was present carrying the reference allele (A) compared to when no 3'UTR was present (3'UTR -), showing that miR-215 represses gene expression by interacting with the 3'UTR when the reference allele 'A' is present. Introducing the alternative ‘C’ allele led to a significant increase in reporter gene expression, reflecting a loss of mir-215 regulation. This is further confirmed since introducing the ‘C’ allele was equivalent to complete deletion of the mir-215 binding site (‘DEL’). Significant differences between groups were calculated using an ANOVA test followed by post-hoc Tukey calculation. All pairwise comparisons were significantly different (*p < 0.05), except for between the ‘C’ and ‘DEL’ constructs which were not significantly different, as indicated (NS). All results are reported as the average +/− standard deviation of 3 biological replicates.

**
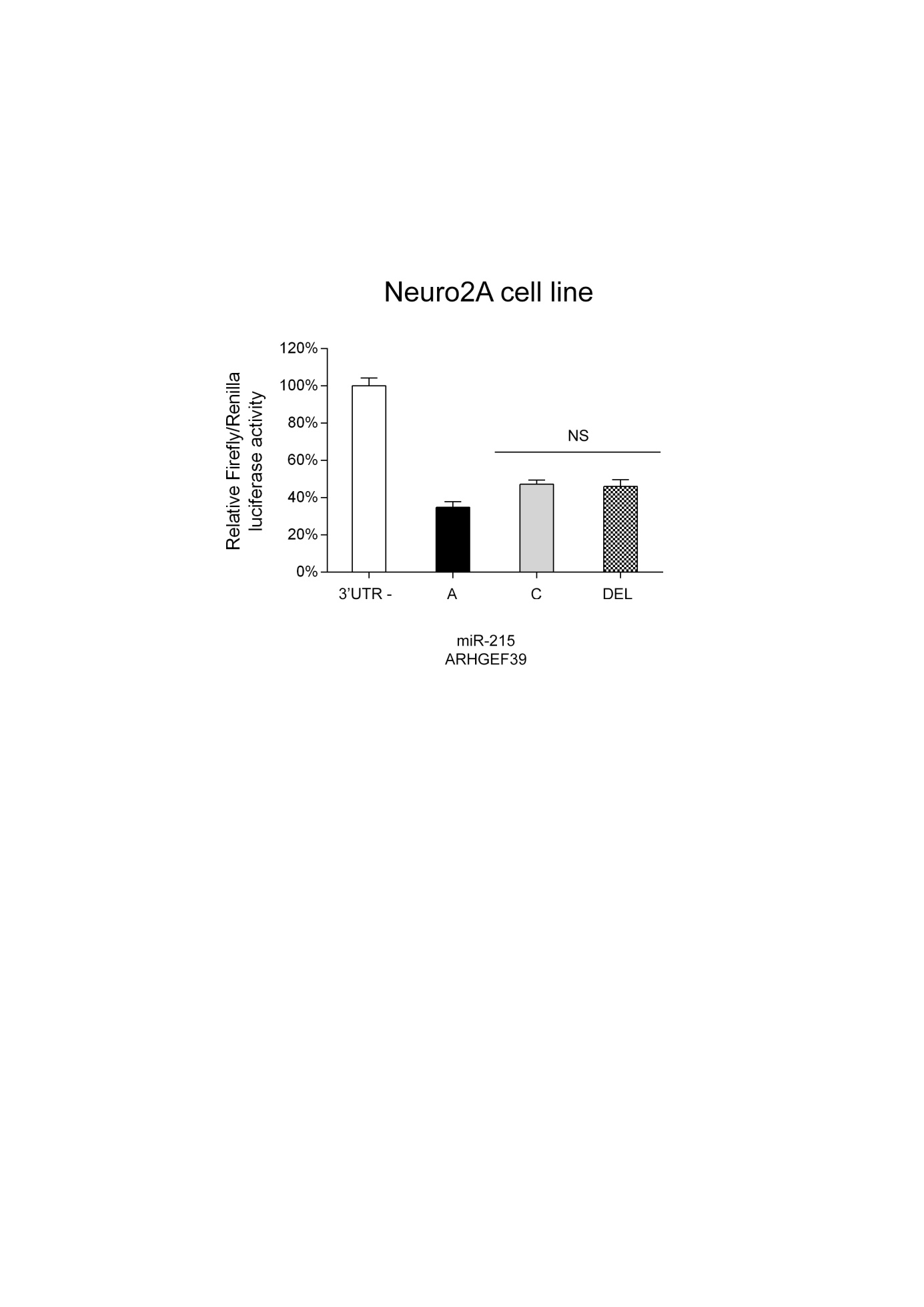
**

**Figure S4. Western blots demonstrating repression of endogenous ARHGEF39 protein by miR-215**. Lysates of cells transfected with miR-215 (+) or an empty vector control (-) were resolved on a 4-15% SDS-PAGE gel and transferred to a PVDF membrane. Proteins were detected via western blotting using antibodies raised to ARHGEF39 or the β-actin loading control. The proteins are observed at their expected sizes of ~38 kDa (ARHGEF39) and ~42 kDa (β-actin).


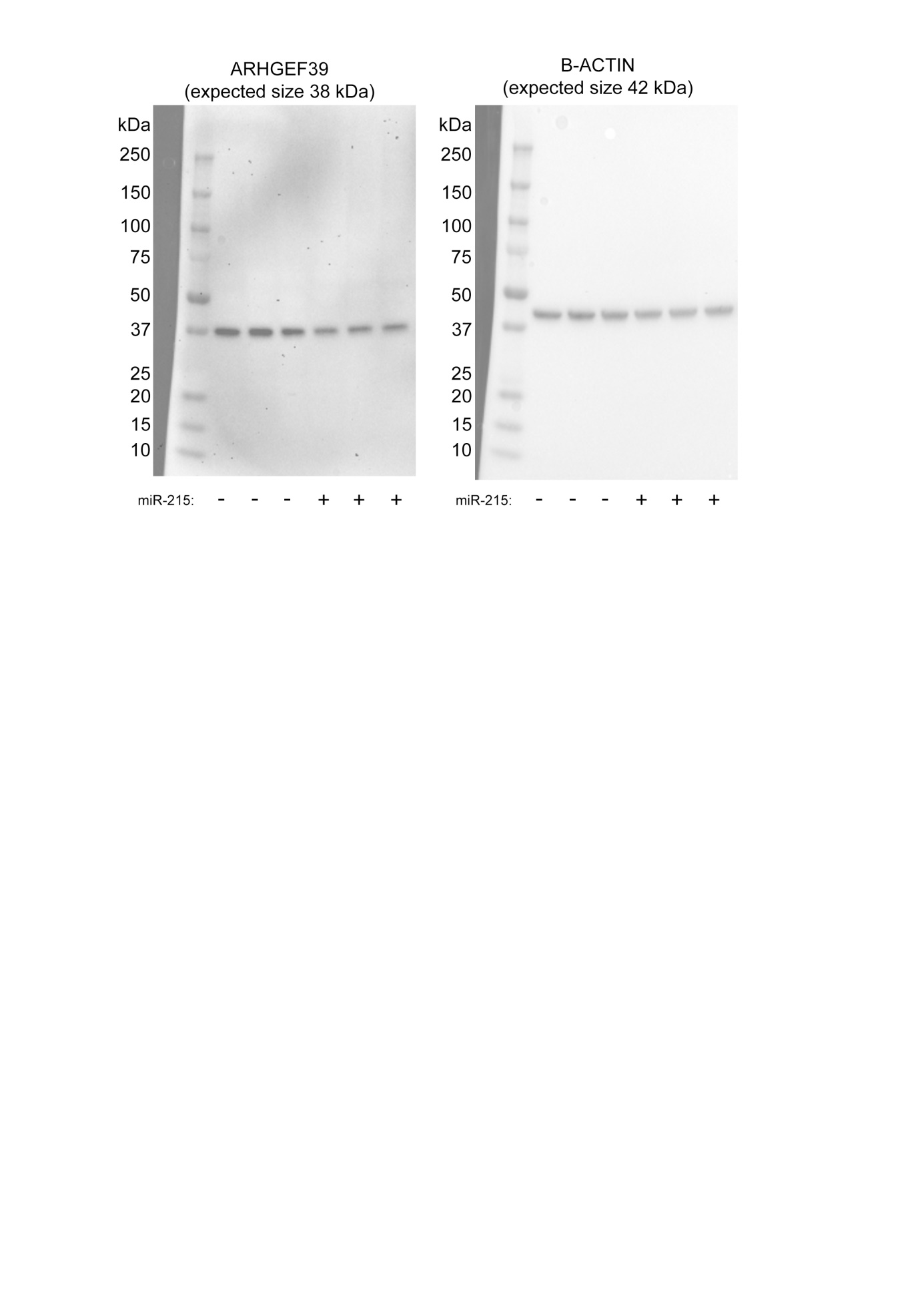


**SUPPLEMENTARY TABLES**

**Table S1 - Summary of the number of quality filtered SNVs in different genomic regions.**

| **Exonic** | **Splicing** | **Intronic** | **5’UTR** | **Up/down-stream** | **3’UTR** | **Intergenic** | **ncRNA** |
| --- | --- | --- | --- | --- | --- | --- | --- |
| **66626** | **430** | **122164** | **2892** | **2870** | **6606** | **46799** | **11881** |

**Table S2 - Association Analyses in SLIC Cohort**

|  | SLIC Discovery Cohort  (983 individuals genotyped) | | | | |
| --- | --- | --- | --- | --- | --- |
| SNP^a^ | ELS (n=467) | RLS (n=465) | NWR (n=706) | spelling (n=290) | reading (n=294) |
| rs72727021 | 0.10 | 0.09 | **0.0077** | 0.56 | 0.20 |
| rs383362 | 0.56 | 0.94 | 0.20 | 0.10 | 0.08 |
| rs1049232 | 0.26 | 0.63 | 0.50 | 1.00 | 1.00 |
| rs1054528 | na | na | na | na | na |
| rs190191374 | na | na | na | na | na |

QFAM test of association was performed in PLINK with an adaptive permutation for all traits shown.

a - rs72727021 was imputed in the SLIC cohort, rs383362 and rs1049232 were directly genotyped as part of a genome-wide SNP analysis. No genotypes were available for rs1054528 and rs190191374.

All p-values shown are empirical.

**Table S3. Cloning and mutagenesis primers with restriction site tags highlighted in BOLD. Gaps or mutated bases introduced via site directed mutagenesis (SDM) are underlined.**

| **3' UTR** | **FORWARD PRIMER (5’→3’)** | **REVERSE PRIMER (5’→3’)** |
| --- | --- | --- |
| **BTN2A1** | **TCTAGA**ACAGCCATGTAGACAAGCCC | **GGCCGGCC**TTCCCTCTCCTCTCTGGTGG |
| **ARHGEF39** (**C9ORF100)** | **TCTAGA**CCACAGCAGCCAGAAAAACT | **GGCCGGCC**TCACTGTGTTGCCCAGGCTG |
| **CENPJ** | **TCTAGA**ACACGGAGCTGTGACGATCCTCAT | **GGCCGGCC**ACAAGTCCAGTGCTCTACGGCT |
| **MTMR3** | **TCTAGA**CCAACTGAAGCTCAGTGACCTGG | **GGCCGGCC**TTTCTTCCCTGCTCACGGTCTCA |
| **3' UTR SDM** | **SENSE OLIGO (5’→3’)** | **ANTISENSE OLIGO (5’→3’)** |
| **ARHGEF39**  **(C9ORF100)**  **(variant)** | GAACTCAGGATACCTCAGGGAGAGGTCACAGCC | GGCTGTGACCTCTCCCTGAGGTATCCTGAGTTC |
| **ARHGEF39**  **(C9ORF100)**  **(deletion)** | TCAGGATACCTCAGGGA_______CAGCCAAGAGTACAAAG | CTTTGTACTCTTGGCTG_______TCCCTGAGGTATCCTGA |
| **miRNAs** | **FORWARD PRIMER (5’→3’)** | **REVERSE PRIMER (5’→3’)** |
| **miR-215** | **ACCGGT**GGACTCTCATTTGATTCCAGCAG | **GAATTC**CCTGCCATTGCGTTGGTTGCGT |
| **miR-342** | **ACCGGT**CCTCTCATAGCCTGGCACTTCC | **GAATTC**CAGTATGCACACCTCGGGGC |
| **miR-346** | **ACCGGT**TTGGCTGCAGGTTGGAGGGCTT | **GAATTC**GTAGGTTGGGAGCGAAGTGGTG |
| **miR-433** | **TCTAGA**CCGGGGAGAAGTACGGTGAGCCTG | **GTCGAC**CCTGGAGAACACCGAGGAGCCCATC |
| **miRNAs POSITIVE CONTROL REPORTERS** | **SENSE OLIGO (5’→3’)** | **ANTISENSE OLIGO (5’→3’)** |
| **miR-215-5p-Reporter** | **GACCC**GTCTGTCAACCATAGGTCATATATTCGTCTGTCAACCATAGGTCAT**GG** | **GTCCC**ATGACCTATGGTTGACAGACGAATATATGACCTATGGTTGACAGAC**GG** |
| **miR-342-3p-Reporter** | **GACCC**ACGGGTGCGATTTAGTGTGAGAATATTCACGGGTGCGATTTAGTGTGAGA**GG** | **GTCCC**TCTCACACTAAATCGCACCCGTGAATATTCTCACACTAAATCGCACCCGT**GG** |
| **miR-346-Reporter** | **GACCC**AGAGGCAGGCCACGGGCAGACAATATTCAGAGGCAGGCCACGGGCAGACA**GG** | **GTCCC**TGTCTGCCCGTGGCCTGCCTCTGAATATTGTCTGCCCGTGGCCTGCCTCT**GG** |
| **miR-433-3p-Reporter** | **GACCC**ACACCGAGGAGCAATCATGATATATTCACACCGAGGAGCAATCATGAT**GG** | **GTCCC**ATCATGATTGCTCCTCGGTGTGAATATATCATGATTGCTCCTCGGTGT**GG** |
